# Metabolomics for Prediction of Relapse in Graves' Disease: Observational Pilot Study

**DOI:** 10.3389/fendo.2018.00623

**Published:** 2018-10-17

**Authors:** Tristan Struja, Andreas Eckart, Alexander Kutz, Andreas Huber, Peter Neyer, Marius Kraenzlin, Beat Mueller, Christian Meier, Luca Bernasconi, Philipp Schuetz

**Affiliations:** ^1^Division of Endocrinology, Diabetes and Metabolism, Medical University Department, Kantonsspital Aarau, Aarau, Switzerland; ^2^Department of Laboratory Medicine, Kantonsspital Aarau, Aarau, Switzerland; ^3^Endonet, Basel, Switzerland; ^4^Medical Faculty, University of Basel, Basel, Switzerland

**Keywords:** Graves basedow disease, metabolomics, relapse activity, predicable results, retrospective analysis

## Abstract

**Background:** There is a lack of biochemical markers for early prediction of relapse in patients with Graves' disease [GD], which may help to direct treatment decisions. We assessed the prognostic ability of a high-throughput proton NMR metabolomic profile to predict relapse in a well characterized cohort of GD patients.

**Methods:** Observational study investigating patients presenting with GD at a Swiss hospital endocrine referral center and an associated endocrine outpatient clinic. We measured 227 metabolic markers in the blood of patients before treatment initiation. Main outcome was relapse of hyperthyroidism within 18 months of stopping anti-thyroid drugs. We used ROC analysis with AUC to assess discrimination.

**Results:** Of 69 included patients 18 (26%) patients had a relapse of disease. The clinical GREAT score had an AUC of 0.68 (95% CI 0.63–0.70) to predict relapse. When looking at the metabolomic markers, univariate analysis revealed pyruvate and triglycerides in medium VLDL as predictors with AUCs of 0.73 (95% CI 0.58–0.84) and 0.67 (95% CI 0.53–0.80), respectively. All other metabolomic markers had lower AUCs.

**Conclusion:** Overall, metabolomic markers in our pilot study had low to moderate prognostic potential for prediction of relapse of GD, with pyruvate and triglycerides being candidates with acceptable discriminatory abilities. Our data need validation in future larger trials.

## Introduction

Graves' disease [GD] is among the leading causes of hyperthyroidism affecting approximately 0.5% of the general population, especially younger women. It is caused by the presence of autoantibodies to the thyrotropin [TSH] receptor [TRAb] leading to unregulated production and secretion of thyroid hormones (1).

Although treatment with thyroidectomy or radioactive iodine ablation [RAI] provide good cure rates from hyperthyroidism, they are definitive ablative procedures rendering patients subject to lifelong therapy with levothyroxine [T4] (2). On the other hand, anti-thyroid drugs [ATD] provide the chance of cure, albeit, at the cost of a very high relapse rate of approximately 40-60% (1). A more personalized approach would include identifying those who were to benefit most of ATD therapy before treatment initiation. Various approaches have been studied in the past, such as genome wide association studies, thyroidal blood flow assessed by sonography, numerous TRAb assays, and combinations of biochemical and epidemiological markers (3, 4). So far, none have provided enough predictive power to be widely adopted into clinical practice.

Recently, the concept of extensively mapping the phenotypic metabolic state of an individual (i.e., metabolome) has become available by advances in spectrometric techniques. Some studies have already mapped the metabolic differences of hyperthyroid GD states compared to euthyroidism (5, 6).

In other areas, predictive qualities of metabolomics have already been assessed. One report showed that inclusion of lysophosphatidylcholine (20:4) as marker improved recurrence risk prediction of strokes by 6% (7), whereas another report found elevated levels of decanoylcarnitine and octanoylcarnitine to be associated with a higher stroke recurrence risk (hazard ratios 3.8 and 5.5, respectively) (8). Such findings have also been observed in a rat model of ANCA positive vasculitis (immunized to human myeloperoxidase), where urinary di-methyl-glycine and trimethylamine N-oxide levels at day 56 post immunization increased relapse prediction accuracy from 90.5 to 95.2% (9). Furthermore, a Japanese group measured plasma free amino acids in patients with ulcerative colitis. They observed that lower levels of histidine were associated with an increased risk of relapse within year (10).

We hypothesized that distinct metabolic patterns might predict outcome of ATD therapy with regard to relapse. To our knowledge, this study is the first to assess whether metabolomic differences can be used to predict relapse of hyperthyroidism after a course of ATD.

## Methods

From a previous observational cohort study (11), we had roughly 320 serum aliquots left over at our disposal. Patients were included at an endocrine outpatient clinic and one hospital-based referral center in Switzerland. Patients were treated with ATD in a titration regimen (usually carbimazole or propylthiouracil for 12-18 months). Inclusion criteria were a first episode of GD defined as suppressed TSH (<0.01 mU/l), elevated free T4, and if available, diffuse increased uptake in scintigraphy. Patients with a shorter follow-up period than 24 months after start of ATD treatment were excluded. Also, we excluded patients with ATD treatment duration <12 months, initial ablative therapy (i.e., surgery or radio-active iodine), and time gap between initiation of treatment and blood sample collection over one month. Aforementioned aliquots were analyzed in the current study. After application of the inclusion and exclusion criteria, there were 69 patients left for final analysis. We collected clinical data by medical charts review and if necessary we complemented missing follow-up data by phone calls to patients and general practitioners. The study protocol was approved by the local ethics committee (Ethikkommission Nordwest- und Zentralschweiz (EKNZ) Project No. 2015/227) and has been conducted according to the principles of the Declaration of Helsinki. Need for informed consent was waived due to retrospective nature of analysis with no impact on health outcome.

After blood withdrawal, samples were directly centrifuged and analyzed on serum TSH, fT4, anti-Thyroperoxidase-Antibodies [anti-TPO-Ab] and TRAb levels by standard commercially available laboratory kits (Assays used, are listed in Supplementary Table 1). Leftover serum aliquots were stored at−24° Celsius and mean duration storage time was 46 months (median 46 months; 70 to 17 months interquartile range). Two hundred and twenty-seven metabolic biomarkers were quantified from serum using high-throughput proton NMR metabolomics (Nightingale Health Ltd., Helsinki, Finland) (12, 13) (biomarkers assessed are listed in Supplementary Table 2). This technique is able to provide excellently reproducible results and analytical accuracy given its limitations regarding sensitivity and resolution as an NMR based method (14, 15). Aliquots were shipped on dry ice by a professional courier service and temperature inside the box was monitored continuously.

To validate our storage conditions and the quality of our samples, Nightingale compared our data with their reference data (see Supplementary Figure 1). Reference values were derived from several studies in Scandinavian and UK cohorts with adjacent biobanks [e.g., most recent publication with references to previous works (16)], mainly from the Finnish National Institute of Health and Welfare Biobanks (THL) (17).

Prior to statistical analysis, data was cube root transformed, normalized by the median of each sample, and Pareto scaled to achieve a normal distribution.

The primary outcome of this study was prediction of relapse in GD at ATD treatment initiation. Relapse had to be established by suppressed TSH and elevated peripheral hormones. First, we fitted univariate ROC models for every metabolomic marker. Second, multivariate ROC models were fit by Monte-Carlo cross validation using balanced sub-sampling. We used partial least squares discriminant analysis [PLS-DA] as classification and feature ranking method. Each cross-validation used two thirds of the samples to gauge feature importance. Top important features where then used to build classification models by using the remaining third of samples (18). To account for multiple testing, correction with Benjamini–Hochberg false discovery rate was applied. Statistical significance was set at α < 0.05. Statistical analysis was conducted using MetaboAnalyst software version 4.0 (19, 20) and Stata software version 12.1 (Stata Corp., College Station, TX, USA).

## Results

Table 1 shows details of the patient population stratified by relapse, the primary endpoint. Previously, we published a validation study of the GREAT score, a combination of epidemiolocal (i.e., age, goiter size) and standard laboratory variables (i.e., fT4, TRAb) to predict relapse (11). It showed an AUC of 0.68 (95% CI 0.63–0.70) to predict relapse.

**Table 1:** Baseline characteristics according to relapse status.

		**No relapse**	**Relapse**
		***N*** = **51**	***N*** = **18**
Sex (F/M)	F	41 (80%)	15 (83%)
	M	10 (20%)	3 (17%)
Age (years)		51 ± 13	47± 13
BMI (kg/m^2^)		24 ± 4.2	23 ± 2.8
Treatment time (months)		20 (18–22)	19 (18–21)
Follow-up after ATD withdrawal (months)		11 (3.9–28)	1 (0.5–12)
Thyroid volume by sonography (mL)		14 (11–16)	15 (9.8–17)
Goiter size (struma grade, 0-III)	0	24 (56%)	9 (60%)
	I	10 (23%)	5 (33%)
	II	8 (19%)	1 (7%)
	III	1 (2%)	0 (0%)
	Missing	8	3
Orbitopathy		13 (25%)	7 (39%)
Smoking		8 (16%)	1 (6%)
fT4 (pM)		30 (21–36)	38 (21–55)
T3 (pM)		3.5 (2.3–4.3)	2.9 (2.8–7.1)
TPO-AK (U/L)		91 (34–454)	163 (90–357)
TRAb (U/L)		5.2 (2.6–11)	12 (3.5–27)
Additional autoimmune diseases	GIT (IBD, celiac disease, pernicious anemia)	1	1
	Type I Diabetes mellitus	1	0
	Other	1	0

*Data presented as counts (percentages), mean (± standard deviation), or median (interquartile range). ATD, anti-thyroid drugs; GIT, gastrointestinal tract; IBD, inflammatory bowel disease; pM, pmol/L*.

Comparison of our data with the reference data set revealed relevant differences for omega-3, glutamine, pyruvate, citrate and acetate. Minor differences were observed for phosphoglycerides, phosphatidylcholines, total cholines, unsaturated fatty acids, and VLDL and LDL diameter, but not in HDL diameter (see Supplementary Figure 1).

Univariate analysis only revealed pyruvate and triglycerides in medium VLDL [MVLDLTG] as significant predictors of GD relapse with AUCs of 0.73 (95% CI 0.58–0.84) and 0.67 (95% CI 0.53–0.80), respectively.

**Figure 1:**
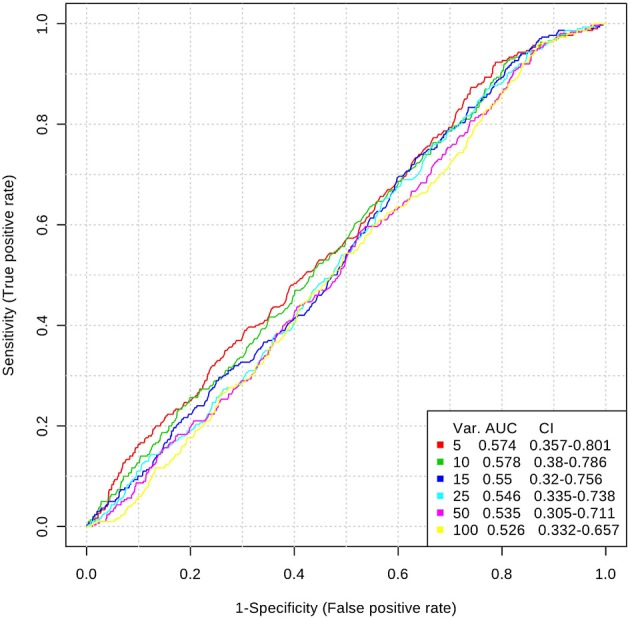
Top 6 ROC models generated by PLS-DA with increasing number of variables. AUC, area under the curve; CI, 95% confidence intervals; PLS-DA, partial least squares-discriminant analysis; Var, number of variables included into model.

**Figure 2:**
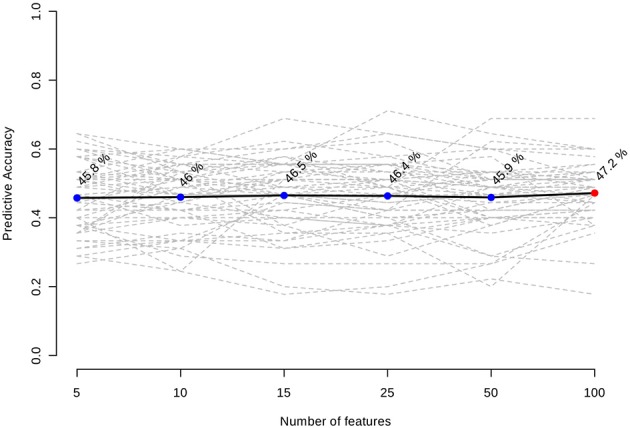
Predictive accuracies of the models with increasing number features included.

**Figure 3:**
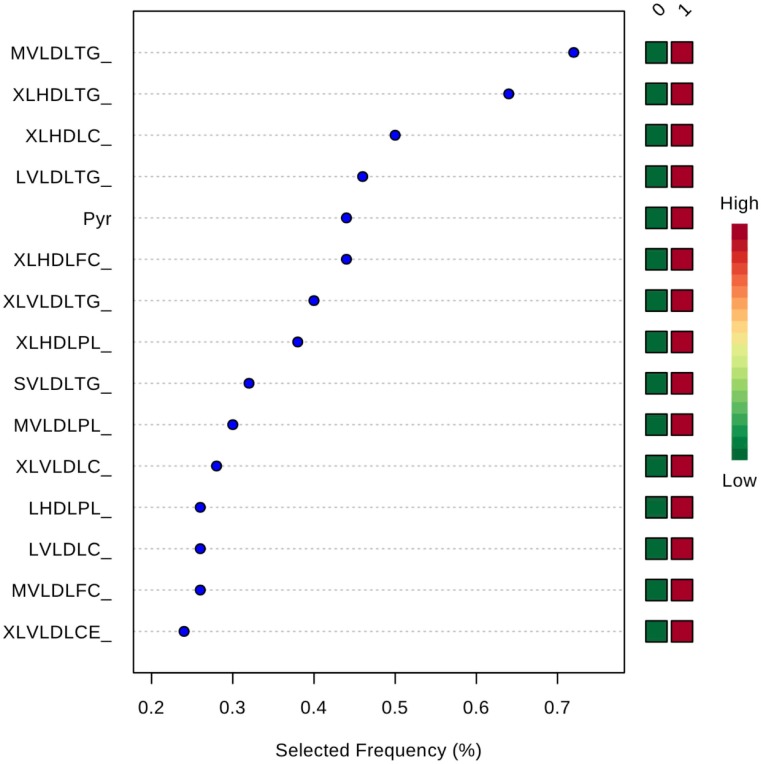
Frequency of a variable being selected by PLS-DA. MVLDLTG, triglycerides in medium VLDL; XLHDLTG, triglycerides in very large HDL; XLHDLC, total cholesterol in very large HDL; LVLDLTG, triglycerides in chylomicrons and extremely large VLDL; Pyr, pyruvate; XLHDLFC, free cholesterol in very large HDL; XLVLDLTG, triglycerides in chylomicrons and extremely large VLDL; XLHDLPL, phospholipids in very large HDL; SVLDLTG, triglycerides in small VLDL; MVLDLPL, phospholipids in medium VLDL; XLVLDLPL, phospholipids in chylomicrons and extremely large VLDL; LHDLPL, phospholipids in very large HDL; LVLDLC, total cholesterol in chylomicrons and extremely large VLDL; MVLDLFC, free cholesterol in medium VLDL; XLVLDLCE, cholesterol esters in chylomicrons and extremely large VLDL.

Inclusion of multiple variables by multivariate ROC analysis did not yield higher AUCs. Figure 1 provides an overview of the top six models generated. AUCs ranged from 0.53 (95% CI 0.33–0.66; 100 variables) to 0.57 (95% CI 0.36–0.80; 5 variables), each not being statistically significant. Inclusion of more variables into a model did not result in improved discriminatory power (see Figure 2). Figure 3 displays the frequency of a variable being selected by PLS-DA.

As there were no significant results although PLS-DA tends to overfit data, we abstained from validating the model in a subset of our data.

## Discussion

Based on this observational, secondary analysis of blood samples, we were not able to find any metabolomic markers that could predict relapse outcome before ATD treatment initiation with high accuracy. To the best of our knowledge, we are the first to apply the principle of metabolomic phenotyping on relapse prediction in GD.

Although we measured roughly 300 samples, we decided to generate a homogenous cohort by applying stringent inclusion and exclusion criteria leading to many exclusions. We did loosen our exclusion criteria *post-hoc* to include more patients, but this did not influence results in any way.

Our model was not able to generate any predictive properties which is reflected by the AUCs around 0.55. Inclusion of more variables into a ROC model usually leads to better predictive capacities at the cost of decreasing practicability (20). In our case, AUC tended to decrease with a growing number of variables in a model. We assume this is a chance finding as all median values are very close to each other, and CIs do overlap.

While there are already some reports investigating the metabolomic phenotype of hyperthyroid GD patients (5, 6, 21, 22). Not surprisingly, there were distinct differences in metabolic pathways between the euthyroid and hyperthyroid state detected. Besides histamine and nitrogen pathways, amino acid pathways were mainly involved. For instance, Piras et al. reported the changes from the hyperthyroid to euthyroid state in 15 patients with GD compared to 26 healthy controls (22). They found that GD patients after treatment had significantly lower levels of creatinine, formate, glycerol, histamine, methylamine, and methylsuccinate in plasma as compared to the healthy controls.

Al-Majdoub and colleagues reported changes in the carnitine metabolism of 30 GD patients before treatment compared to 12 months after institution of euthyroidism (5). They observed an increase in short-chain acylcarnitines, whereas medium-chain acylcarnitines were decreased and long-chain acylcarnitines were unchanged after treatment. In general, lysophosphatidylcholines and sphingomyelins were increased in their study. The authors speculated that these changes reflect a starvation like process that was induced by hyperthyroidism.

In 2016, researchers from Singapore published their data on 24 female GD patients transitioning from hyperthyroidism to euthyroidism. In contrast to the previous report, they found a fall of medium- and long-chain acylcarnitines, whereas they observed rises in total cholesterol, LDL, and HDL. The authors postulate that the changes in cholesterol metabolism might be due to increased clearance in hyperthyroidism, whereas the changes in acylcarnitines is based on the T3 induced increased mitochondrial biogenesis and enhanced tricarboxylic acid cycle activity. They also found no changes in branched chain amino acid concentrations (i.e., valine, isoleucine, and leucine). On the other hand, levels of phenylalanine and tyrosine were elevated which might have been due to the increased demand of these amino acid in the synthesis of thyroid hormones (6).

So far there was no investigation looking at the relation to relapse rates. Thus, we studied all markers for their potential to predict relapse with the risk for chance findings. Our data are thus rather hypothesis-generating and need to be validated in future studies. Compared to the previous studies, our laboratory assay put more emphasis on lipid pathways but not carnitine and amino acid metabolism, which might explain our negative findings (see Supplementary Figure 1 and Supplementary Table 2) or it could be due to our limitations in study design. As our focus was prediction of relapse and had only blood samples before the start of treatment, we did not investigate metabolic changes during the transition from hyperthyroidism to euthyroidism.

Our study has three major limitations. First, blood samples were not drawn in a fasting state but randomly. Second, mean storage time of samples was 46 months under sub-optimal conditions (i.e., −24°C instead of −80°C) (23), although other groups reported significant results after storage at −24°C (5). On one hand, suboptimal storage conditions lead to low levels of glutamine, phenylalanine, pyruvate, and acetate. On the other hand, these metabolites are very scarce and at least for lipid components a large coefficient of variation has been reported even under optimal conditions (24). Moreover, glycerol, lactate, and creatinine which are shown to be altered significantly by freeze-thaw cycles in rats (25), have not shown obvious deviations in our cohort.

Furthermore, we did not observe large deviations from the reference sample in fatty acids, especially polyunsaturated fatty acids, which would be other indicators of suboptimal storage and handling (24). Additionally, metabolites such as glucose and lactate that are known to be susceptible to preanalytical errors and have been proposed as a screening tool to assess preanalytical care (26). In our batch, these metabolites were not altogether different from the manufacturer's reference sample.

Third, we had two freeze-thaw cycles during our sample preparation before analysis. From an idealistic standpoint, immediate analysis after blood draw would be the preferable approach which is rarely feasible in routine. Furthermore, a report demonstrated that up to four freeze-thaw cycles did alter samples only slightly (23).

## Conclusion

Overall, metabolomic markers in our pilot study had only low to moderate prognostic potential for prediction of relapse of GD, with pyruvate and triglycerides being the most promising candidates with acceptable discriminatory ability. Our data need validation in future larger trials.

## Author contributions

TS analyzed data and wrote the first draft of the manuscript with primary responsibility for the final content. All authors read and approved the final manuscript.

### Conflict of interest statement

The authors declare that the research was conducted in the absence of any commercial or financial relationships that could be construed as a potential conflict of interest.
