# Supplementary material for: Metabolomics for Prediction of Relapse in Graves' Disease: Observational Pilot Study
Source: Front Endocrinol (Lausanne). 2018 Oct 17;9:623. doi: 10.3389/fendo.2018.00623 (PMC6199355; doi:10.3389/fendo.2018.00623)
Supplement: Supplementary file 1 [file Data_Sheet_1.docx]

**Metabolomics for prediction of relapse in Graves’ disease: observational pilot study**

Tristan Struja^1^, Andreas Eckart^1^, Alexander Kutz^1^, Andreas Huber^2^, Marius Kraenzlin^4^, Beat Mueller^1,3^, Christian Meier^3,4^, ^†^Luca Bernasconi^2^, and ^†^Philipp Schuetz^1,3^

^†^equally contributing senior authors

[tristan.struja@gmail.com](mailto:tristan.struja@gmail.com); eckartan@gmail.com; kutz.alexander@gmail.com; andreas.huber@ksa.ch; marius.kraenzlin@unibas.ch; [christian.meier@unibas.ch](mailto:christian.meier@unibas.ch); [happy.mueller@unibas.ch](mailto:happy.mueller@unibas.ch); [luca.bernasconi@ksa.ch](mailto:luca.bernasconi@ksa.ch); [schuetzph@gmail.com](mailto:schuetzph@gmail.com)

^1^ Medical University Department, Division of Endocrinology, Diabetes & Metabolism, Kantonsspital Aarau, Aarau, Switzerland; ^2^ Department of Laboratory Medicine, Kantonsspital Aarau, Switzerland; ^3^ Medical Faculty of the University of Basel, Switzerland; ^4^ Endonet, Basel, Switzerland

SUPPLEMENTARY MATERIAL: 2 tables, 1 figure

**Corresponding author and person to whom reprint requests should be addressed:**

Tristan Struja, MD

University Dept. of Medicine, Division of Endocrinology, Diabetes, and Metabolism, Kantonsspital Aarau

Tellstrasse, CH-5001 Aarau, Switzerland

tristan.struja@gmail.com (E-Mail)

**Supplementary table 1 Specifications of routine assays used**

|  | **Kantonsspital Aarau** | **Endonet Outpatient Clinic** |
| --- | --- | --- |
| **Timeframe 2010-2014** | | |
| **TSH** | After 01/2011 Dimension Vista, Siemens, Germany  Up to 12/2010 Immulite-TSH, Siemens, Germany | Elecsys TSH Test, Cobas e411, Roche, Switzerland  Coefficient of variation 1.5-8.6%  Lower limit of detection 0.014 U/l |
| **fT4** | After 01/2011 Dimension Vista fT4, Siemens, Germany  Up to 12/2010 Immulite-fT4, Siemens, Germany | Elecsys fT4 Test, Cobas e411, Roche, Switzerland  Coefficient of variation 1.3-4.0%  Lower limit of detection 0.3 pmol/l |
| **fT3** | After 01/2011 Dimension Vista, Siemens, Germany  Up to 12/2010 Immulite-fT3, Siemens, Germany | N/A |
| **T3** | Immulite-T3, Siemens, Germany | Elecsys T3 Test, Cobas e411, Roche, Switzerland  Coefficient of variation 3.6-5.3%  Lower limit of detection 0.3 nmol/l |
| **TPO-Ab** | ELISA, INOVA Diagnostics, USA | Elecsys TPO-Ab Test, Cobas e411, Roche, Switzerland  Coefficient of variation 4.2-7.0%  Lower limit of detection <5.0 U/l |
| **TRAb** | RSR ELISA (3^rd^ generation), RSR Ltd., UK  Cut-Off < 0.4 U/l  Lower limit of detection 0.4U/l | BRAHMS TRAK human LIA, ThermoScientific, Germany  Coefficient of variation 20% 0.8-1.2U/l  Cut- off < 0.4U/l  Lower limit of detection 0.4U/l |
| **Timeframe 2006-2010** | | |
| **TSH** | Immulite-TSH, Siemens, Germany | Elecsys 2010, Roche, Switzerland  Coefficient of variation 1.5-8.6%  Lower limit of detection 0.014 U/l |
| **fT4** | Immulite-fT4 Siemens, Germany | Elecsys 2010, Roche, Switzerland  Coefficient of variation 1.3-4.0%  Lower limit of detection 0.3 pmol/l |
| **fT3** | Immulite-fT3, Siemens, Germany | N/A |
| **T3** | Immulite-T3, Siemens, Germany | Elecsys 2010, Roche, Switzerland  Coefficient of variation 3.6-5.3%  Lower limit of detection 0.3 nmol/l |
| **TPO-Ab** | ELISA, INOVA Diagnostics, USA | Elecsys 2010, Roche, Switzerland  Coefficient of variation 4.2-7.0%  Lower limit of detection <5.0 U/l |
| **TRAb** | RSR ELISA (2^nd^ generation), RSR Ltd., UK  Cut-Off < 1.8 U/l  Lower limit of detection 0.4U/L | BRAHMS TRAK human LIA, ThermoScientific, Germany  Coefficient of variation 20% 0.8-1.2U/l  Cut- off < 0.4U/l  Lower limit of detection 0.4U/l |
| **Timeframe 2004-2006** | | |
| **TSH** | Immulite-TSH, Siemens, Germany | Elecsys 2010, Roche, Switzerland  Coefficient of variation 1.5-8.6%  Lower limit of detection 0.014 U/l |
| **fT4** | Immulite-fT4 Siemens, Germany | Elecsys 2010, Roche, Switzerland  Coefficient of variation 1.3-4.0%  Lower limit of detection < 0.3 pmol/l |
| **fT3** | Immulite-fT3, Siemens, Germany | N/A |
| **T3** | Immulite-T3, Siemens, Germany | Elecsys 2010, Roche, Switzerland  Coefficient of variation 3.6-5.3%  Lower limit of detection 0.3 nmol/l |
| **TPO-Ab** | ELISA, INOVA Diagnostics, USA | Elecsys 2010, Roche, Switzerland  Coefficient of variation 4.2-7.0%  Lower limit of detection <5.0 U/l |
| **TRAb** | RSR ELISA (2^nd^ generation), RSR Ltd., UK  Cut-Off < 1.8 U/l  Lower limit of detection 0.4U/L | BRAHMS TRAK human LIA, ThermoScientific, Germany  Coefficient of variation 20% 0.8-1.2U/l  Cut- off < 0.4U/l  Lower limit of detection < 0.4U/l |

**Supplementary table 2 List of analytes measured by NMR**

| **Analyte** | **Abbr.** | **Unit** |
| --- | --- | --- |
| Concentration of chylomicrons and extremely large VLDL particles | XXL-VLDL-P | mol/l |
| Total lipids in chylomicrons and extremely large VLDL | XXL-VLDL-L | mmol/l |
| Phospholipids in chylomicrons and extremely large VLDL | XXL-VLDL-PL | mmol/l |
| Total cholesterol in chylomicrons and extremely large VLDL | XXL-VLDL-C | mmol/l |
| Cholesterol esters in chylomicrons and extremely large VLDL | XXL-VLDL-CE | mmol/l |
| Free cholesterol in chylomicrons and extremely large VLDL | XXL-VLDL-FC | mmol/l |
| Triglycerides in chylomicrons and extremely large VLDL | XXL-VLDL-TG | mmol/l |
| Concentration of very large VLDL particles | XL-VLDL-P | mol/l |
| Total lipids in very large VLDL | XL-VLDL-L | mmol/l |
| Phospholipids in very large VLDL | XL-VLDL-PL | mmol/l |
| Total cholesterol in very large VLDL | XL-VLDL-C | mmol/l |
| Cholesterol esters in very large VLDL | XL-VLDL-CE | mmol/l |
| Free cholesterol in very large VLDL | XL-VLDL-FC | mmol/l |
| Triglycerides in very large VLDL | XL-VLDL-TG | mmol/l |
| Concentration of large VLDL particles | L-VLDL-P | mol/l |
| Total lipids in large VLDL | L-VLDL-L | mmol/l |
| Phospholipids in large VLDL | L-VLDL-PL | mmol/l |
| Total cholesterol in large VLDL | L-VLDL-C | mmol/l |
| Cholesterol esters in large VLDL | L-VLDL-CE | mmol/l |
| Free cholesterol in large VLDL | L-VLDL-FC | mmol/l |
| Triglycerides in large VLDL | L-VLDL-TG | mmol/l |
| Concentration of medium VLDL particles | M-VLDL-P | mol/l |
| Total lipids in medium VLDL | M-VLDL-L | mmol/l |
| Phospholipids in medium VLDL | M-VLDL-PL | mmol/l |
| Total cholesterol in medium VLDL | M-VLDL-C | mmol/l |
| Cholesterol esters in medium VLDL | M-VLDL-CE | mmol/l |
| Free cholesterol in medium VLDL | M-VLDL-FC | mmol/l |
| Triglycerides in medium VLDL | M-VLDL-TG | mmol/l |
| Concentration of small VLDL particles | S-VLDL-P | mol/l |
| Total lipids in small VLDL | S-VLDL-L | mmol/l |
| Phospholipids in small VLDL | S-VLDL-PL | mmol/l |
| Total cholesterol in small VLDL | S-VLDL-C | mmol/l |
| Cholesterol esters in small VLDL | S-VLDL-CE | mmol/l |
| Free cholesterol in small VLDL | S-VLDL-FC | mmol/l |
| Triglycerides in small VLDL | S-VLDL-TG | mmol/l |
| Concentration of very small VLDL particles | XS-VLDL-P | mol/l |
| Total lipids in very small VLDL | XS-VLDL-L | mmol/l |
| Phospholipids in very small VLDL | XS-VLDL-PL | mmol/l |
| Total cholesterol in very small VLDL | XS-VLDL-C | mmol/l |
| Cholesterol esters in very small VLDL | XS-VLDL-CE | mmol/l |
| Free cholesterol in very small VLDL | XS-VLDL-FC | mmol/l |
| Triglycerides in very small VLDL | XS-VLDL-TG | mmol/l |
| Concentration of IDL particles | IDL-P | mol/l |
| Total lipids in IDL | IDL-L | mmol/l |
| Phospholipids in IDL | IDL-PL | mmol/l |
| Total cholesterol in IDL | IDL-C | mmol/l |
| Cholesterol esters in IDL | IDL-CE | mmol/l |
| Free cholesterol in IDL | IDL-FC | mmol/l |
| Triglycerides in IDL | IDL-TG | mmol/l |
| Concentration of large LDL particles | L-LDL-P | mol/l |
| Total lipids in large LDL | L-LDL-L | mmol/l |
| Phospholipids in large LDL | L-LDL-PL | mmol/l |
| Total cholesterol in large LDL | L-LDL-C | mmol/l |
| Cholesterol esters in large LDL | L-LDL-CE | mmol/l |
| Free cholesterol in large LDL | L-LDL-FC | mmol/l |
| Triglycerides in large LDL | L-LDL-TG | mmol/l |
| Concentration of medium LDL particles | M-LDL-P | mol/l |
| Total lipids in medium LDL | M-LDL-L | mmol/l |
| Phospholipids in medium LDL | M-LDL-PL | mmol/l |
| Total cholesterol in medium LDL | M-LDL-C | mmol/l |
| Cholesterol esters in medium LDL | M-LDL-CE | mmol/l |
| Free cholesterol in medium LDL | M-LDL-FC | mmol/l |
| Triglycerides in medium LDL | M-LDL-TG | mmol/l |
| Concentration of small LDL particles | S-LDL-P | mol/l |
| Total lipids in small LDL | S-LDL-L | mmol/l |
| Phospholipids in small LDL | S-LDL-PL | mmol/l |
| Total cholesterol in small LDL | S-LDL-C | mmol/l |
| Cholesterol esters in small LDL | S-LDL-CE | mmol/l |
| Free cholesterol in small LDL | S-LDL-FC | mmol/l |
| Triglycerides in small LDL | S-LDL-TG | mmol/l |
| Concentration of very large HDL particles | XL-HDL-P | mol/l |
| Total lipids in very large HDL | XL-HDL-L | mmol/l |
| Phospholipids in very large HDL | XL-HDL-PL | mmol/l |
| Total cholesterol in very large HDL | XL-HDL-C | mmol/l |
| Cholesterol esters in very large HDL | XL-HDL-CE | mmol/l |
| Free cholesterol in very large HDL | XL-HDL-FC | mmol/l |
| Triglycerides in very large HDL | XL-HDL-TG | mmol/l |
| Concentration of large HDL particles | L-HDL-P | mol/l |
| Total lipids in large HDL | L-HDL-L | mmol/l |
| Phospholipids in large HDL | L-HDL-PL | mmol/l |
| Total cholesterol in large HDL | L-HDL-C | mmol/l |
| Cholesterol esters in large HDL | L-HDL-CE | mmol/l |
| Free cholesterol in large HDL | L-HDL-FC | mmol/l |
| Triglycerides in large HDL | L-HDL-TG | mmol/l |
| Concentration of medium HDL particles | M-HDL-P | mol/l |
| Total lipids in medium HDL | M-HDL-L | mmol/l |
| Phospholipids in medium HDL | M-HDL-PL | mmol/l |
| Total cholesterol in medium HDL | M-HDL-C | mmol/l |
| Cholesterol esters in medium HDL | M-HDL-CE | mmol/l |
| Free cholesterol in medium HDL | M-HDL-FC | mmol/l |
| Triglycerides in medium HDL | M-HDL-TG | mmol/l |
| Concentration of small HDL particles | S-HDL-P | mol/l |
| Total lipids in small HDL | S-HDL-L | mmol/l |
| Phospholipids in small HDL | S-HDL-PL | mmol/l |
| Total cholesterol in small HDL | S-HDL-C | mmol/l |
| Cholesterol esters in small HDL | S-HDL-CE | mmol/l |
| Free cholesterol in small HDL | S-HDL-FC | mmol/l |
| Triglycerides in small HDL | S-HDL-TG | mmol/l |
| Phospholipids to total lipids ratio in chylomicrons and extremely large VLDL | XXL-VLDL-PL_% | % |
| Total cholesterol to total lipids ratio in chylomicrons and extremely large VLDL | XXL-VLDL-C_% | % |
| Cholesterol esters to total lipids ratio in chylomicrons and extremely large VLDL | XXL-VLDL-CE_% | % |
| Free cholesterol to total lipids ratio in chylomicrons and extremely large VLDL | XXL-VLDL-FC_% | % |
| Triglycerides to total lipids ratio in chylomicrons and extremely large VLDL | XXL-VLDL-TG_% | % |
| Phospholipids to total lipids ratio in very large VLDL | XL-VLDL-PL_% | % |
| Total cholesterol to total lipids ratio in very large VLDL | XL-VLDL-C_% | % |
| Cholesterol esters to total lipids ratio in very large VLDL | XL-VLDL-CE_% | % |
| Free cholesterol to total lipids ratio in very large VLDL | XL-VLDL-FC_% | % |
| Triglycerides to total lipids ratio in very large VLDL | XL-VLDL-TG_% | % |
| Phospholipids to total lipids ratio in large VLDL | L-VLDL-PL_% | % |
| Total cholesterol to total lipids ratio in large VLDL | L-VLDL-C_% | % |
| Cholesterol esters to total lipids ratio in large VLDL | L-VLDL-CE_% | % |
| Free cholesterol to total lipids ratio in large VLDL | L-VLDL-FC_% | % |
| Triglycerides to total lipids ratio in large VLDL | L-VLDL-TG_% | % |
| Phospholipids to total lipids ratio in medium VLDL | M-VLDL-PL_% | % |
| Total cholesterol to total lipids ratio in medium VLDL | M-VLDL-C_% | % |
| Cholesterol esters to total lipids ratio in medium VLDL | M-VLDL-CE_% | % |
| Free cholesterol to total lipids ratio in medium VLDL | M-VLDL-FC_% | % |
| Triglycerides to total lipids ratio in medium VLDL | M-VLDL-TG_% | % |
| Phospholipids to total lipids ratio in small VLDL | S-VLDL-PL_% | % |
| Total cholesterol to total lipids ratio in small VLDL | S-VLDL-C_% | % |
| Cholesterol esters to total lipids ratio in small VLDL | S-VLDL-CE_% | % |
| Free cholesterol to total lipids ratio in small VLDL | S-VLDL-FC_% | % |
| Triglycerides to total lipids ratio in small VLDL | S-VLDL-TG_% | % |
| Phospholipids to total lipids ratio in very small VLDL | XS-VLDL-PL_% | % |
| Total cholesterol to total lipids ratio in very small VLDL | XS-VLDL-C_% | % |
| Cholesterol esters to total lipids ratio in very small VLDL | XS-VLDL-CE_% | % |
| Free cholesterol to total lipids ratio in very small VLDL | XS-VLDL-FC_% | % |
| Triglycerides to total lipids ratio in very small VLDL | XS-VLDL-TG_% | % |
| Phospholipids to total lipids ratio in IDL | IDL-PL_% | % |
| Total cholesterol to total lipids ratio in IDL | IDL-C_% | % |
| Cholesterol esters to total lipids ratio in IDL | IDL-CE_% | % |
| Free cholesterol to total lipids ratio in IDL | IDL-FC_% | % |
| Triglycerides to total lipids ratio in IDL | IDL-TG_% | % |
| Phospholipids to total lipids ratio in large LDL | L-LDL-PL_% | % |
| Total cholesterol to total lipids ratio in large LDL | L-LDL-C_% | % |
| Cholesterol esters to total lipids ratio in large LDL | L-LDL-CE_% | % |
| Free cholesterol to total lipids ratio in large LDL | L-LDL-FC_% | % |
| Triglycerides to total lipids ratio in large LDL | L-LDL-TG_% | % |
| Phospholipids to total lipids ratio in medium LDL | M-LDL-PL_% | % |
| Total cholesterol to total lipids ratio in medium LDL | M-LDL-C_% | % |
| Cholesterol esters to total lipids ratio in medium LDL | M-LDL-CE_% | % |
| Free cholesterol to total lipids ratio in medium LDL | M-LDL-FC_% | % |
| Triglycerides to total lipids ratio in medium LDL | M-LDL-TG_% | % |
| Phospholipids to total lipids ratio in small LDL | S-LDL-PL_% | % |
| Total cholesterol to total lipids ratio in small LDL | S-LDL-C_% | % |
| Cholesterol esters to total lipids ratio in small LDL | S-LDL-CE_% | % |
| Free cholesterol to total lipids ratio in small LDL | S-LDL-FC_% | % |
| Triglycerides to total lipids ratio in small LDL | S-LDL-TG_% | % |
| Phospholipids to total lipids ratio in very large HDL | XL-HDL-PL_% | % |
| Total cholesterol to total lipids ratio in very large HDL | XL-HDL-C_% | % |
| Cholesterol esters to total lipids ratio in very large HDL | XL-HDL-CE_% | % |
| Free cholesterol to total lipids ratio in very large HDL | XL-HDL-FC_% | % |
| Triglycerides to total lipids ratio in very large HDL | XL-HDL-TG_% | % |
| Phospholipids to total lipids ratio in large HDL | L-HDL-PL_% | % |
| Total cholesterol to total lipids ratio in large HDL | L-HDL-C_% | % |
| Cholesterol esters to total lipids ratio in large HDL | L-HDL-CE_% | % |
| Free cholesterol to total lipids ratio in large HDL | L-HDL-FC_% | % |
| Triglycerides to total lipids ratio in large HDL | L-HDL-TG_% | % |
| Phospholipids to total lipids ratio in medium HDL | M-HDL-PL_% | % |
| Total cholesterol to total lipids ratio in medium HDL | M-HDL-C_% | % |
| Cholesterol esters to total lipids ratio in medium HDL | M-HDL-CE_% | % |
| Free cholesterol to total lipids ratio in medium HDL | M-HDL-FC_% | % |
| Triglycerides to total lipids ratio in medium HDL | M-HDL-TG_% | % |
| Phospholipids to total lipids ratio in small HDL | S-HDL-PL_% | % |
| Total cholesterol to total lipids ratio in small HDL | S-HDL-C_% | % |
| Cholesterol esters to total lipids ratio in small HDL | S-HDL-CE_% | % |
| Free cholesterol to total lipids ratio in small HDL | S-HDL-FC_% | % |
| Triglycerides to total lipids ratio in small HDL | S-HDL-TG_% | % |
| Mean diameter for VLDL particles | VLDL-D | nm |
| Mean diameter for LDL particles | LDL-D | nm |
| Mean diameter for HDL particles | HDL-D | nm |
| Serum total cholesterol | Serum-C | mmol/l |
| Total cholesterol in VLDL | VLDL-C | mmol/l |
| Remnant cholesterol (non-HDL, non-LDL -cholesterol) | Remnant-C | mmol/l |
| Total cholesterol in LDL | LDL-C | mmol/l |
| Total cholesterol in HDL | HDL-C | mmol/l |
| Total cholesterol in HDL2 | HDL2-C | mmol/l |
| Total cholesterol in HDL3 | HDL3-C | mmol/l |
| Esterified cholesterol | EstC | mmol/l |
| Free cholesterol | FreeC | mmol/l |
| Serum total triglycerides | Serum-TG | mmol/l |
| Triglycerides in VLDL | VLDL-TG | mmol/l |
| Triglycerides in LDL | LDL-TG | mmol/l |
| Triglycerides in HDL | HDL-TG | mmol/l |
| Total phosphoglycerides | TotPG | mmol/l |
| Ratio of triglycerides to phosphoglycerides | TG/PG | % |
| Phosphatidylcholine and other cholines | PC | mmol/l |
| Sphingomyelins | SM | mmol/l |
| Total cholines | TotCho | mmol/l |
| Apolipoprotein A-I | ApoA1 | g/l |
| Apolipoprotein B | ApoB | g/l |
| Ratio of apolipoprotein B to apolipoprotein A-I | ApoB/ApoA1 | % |
| Total fatty acids | TotFA | mmol/l |
| Estimated degree of unsaturation | UnSat | % |
| 22:6, docosahexaenoic acid | DHA | mmol/l |
| 18:2, linoleic acid | LA | mmol/l |
| Omega-3 fatty acids | FAw3 | mmol/l |
| Omega-6 fatty acids | FAw6 | mmol/l |
| Polyunsaturated fatty acids | PUFA | mmol/l |
| Monounsaturated fatty acids; 16:1, 18:1 | MUFA | mmol/l |
| Saturated fatty acids | SFA | mmol/l |
| Ratio of 22:6 docosahexaenoic acid to total fatty acids | DHA/FA | % |
| Ratio of 18:2 linoleic acid to total fatty acids | LA/FA | % |
| Ratio of omega-3 fatty acids to total fatty acids | FAw3/FA | % |
| Ratio of omega-6 fatty acids to total fatty acids | FAw6/FA | % |
| Ratio of polyunsaturated fatty acids to total fatty acids | PUFA/FA | % |
| Ratio of monounsaturated fatty acids to total fatty acids | MUFA/FA | % |
| Ratio of saturated fatty acids to total fatty acids | SFA/FA | % |
| Glucose | Glc | mmol/l |
| Lactate | Lac | mmol/l |
| Pyruvate | Pyr | mmol/l |
| Citrate | Cit | mmol/l |
| Glycerol | Glol | mmol/l |
| Alanine | Ala | mmol/l |
| Glutamine | Gln | mmol/l |
| Glycine | Gly | mmol/l |
| Histidine | His | mmol/l |
| Isoleucine | Ile | mmol/l |
| Leucine | Leu | mmol/l |
| Valine | Val | mmol/l |
| Phenylalanine | Phe | mmol/l |
| Tyrosine | Tyr | mmol/l |
| Acetate | Ace | mmol/l |
| Acetoacetate | AcAce | mmol/l |
| 3-hydroxybutyrate | bOHBut | mmol/l |
| Creatinine | Crea | mmol/l |
| Glycoprotein acetyls, mainly a1-acid glycoprotein | Gp | mmol/l |

**Supplementary figure 1 Distribution of our measurements with the internal reference standard of the laboratory (provided by Nightingale Health Ltd, Helsinki, Finland)**
